# Atypical Presentation of Colonic Sarcoidosis and Current Diagnostic Challenges

**DOI:** 10.1155/crgm/5549338

**Published:** 2025-10-31

**Authors:** Rangesh Modi, Cameron Dandridge, Nada Attia, Edwin McDonald

**Affiliations:** ^1^Department of Internal Medicine, Section of Gastroenterology, Hepatology and Nutrition, University of Chicago Medicine, Chicago 60637, Illinois, USA; ^2^Department of Internal Medicine, University of Chicago Medicine, Chicago 60637, Illinois, USA

## Abstract

We present a case of a Hispanic woman in her 70s who presented with nonspecific lower abdominal pain leading to a diagnosis of hepatic and colonic sarcoidosis. She had elevated liver function tests (LFTs), and imaging ruled out ischemia and cholangitis but revealed a cecal and ascending colon wall thickening with fat stranding. Infectious and autoimmune causes of elevated LFTs were excluded. A liver biopsy showed noncaseating granulomas, indicating hepatic sarcoidosis. Despite no bloody diarrhea, a colonoscopy was performed due to nonspecific abdominal pain and imaging-based evidence of colitis. A colonoscopy revealed diffuse areas of severely erythematous, hyperemic, and ulcerating mucosa in the ascending colon. Biopsies confirmed abundant noncaseating granulomas in the background of inflammation. Stool testing, imaging, and staining of biopsies excluded infectious or ischemic etiologies of colitis. The presence of hepatic sarcoidosis, along with age and symptom profile, prompted a diagnosis of colonic sarcoidosis rather than Crohn's colitis. Prednisone and methotrexate improved her symptoms and LFTs within 3 months of initiation.

## 1. Introduction

Sarcoidosis is a systemic disease with an unknown cause, characterized by the infiltration of various organs by noncaseating epithelioid granulomas. While the lungs are commonly affected, other organs, including the skin, eyes, heart, kidneys, central nervous system, musculoskeletal system, and gastrointestinal (GI) tract, can also be involved. Involvement of the GI tract occurs in less than 2% of the cases. Although the stomach is most commonly affected within the GI tract, reports of sarcoidosis in the small bowel and colon exist in the literature, dating back to 1949. Colonic sarcoidosis typically affects individuals between 30 and 60 years old, presenting with a range of symptoms from asymptomatic polyps discovered during routine colonoscopy to obstructive mass-like lesions with high FDG PET activity, mimicking cancer. Additionally, it can manifest as an ulcerative and inflammatory disease, with biopsies showing noncaseating epithelioid granulomas accompanied by acute or chronic inflammation in the mucosa and submucosa. This presentation can often be mistaken for infectious colitis or inflammatory bowel disease (IBD), such as Crohn's colitis. Clinicians frequently rely on the presence of extraintestinal sarcoidosis, levels of angiotensin-converting enzyme (ACE), and their index of suspicion, which can introduce a high risk of bias. While treatment with steroids is common for both colonic sarcoidosis and IBD, the use of biologics and treatment outcomes, as well as monitoring, can differ significantly. Our case report underscores these diagnostic challenges, detailing the case of an elderly female presenting with abdominal pain and granulomatous colitis due to colonic sarcoidosis.

## 2. Case Presentation

A Hispanic woman in her 70s presented to our tertiary care facility for 8 days of constant aching lower abdominal pain radiating to the left flank, varying intensity from 5/10 to 8/10, with no specific exacerbating or relieving factors. She had mild nausea but no vomiting, diarrhea, or melena. Her past medical history included gastroesophageal reflux disease (GERD) and type 2 diabetes mellitus, and her surgical history included a cholecystectomy done more than 30 years ago. She was a former smoker who quit more than 30 years ago and denied alcohol, marijuana, or illicit drug use. She had no family history of IBD, cancer, or rheumatological diseases. She took 40 mg of pantoprazole daily, metformin 1000 mg twice daily, and 40 insulin-neutral protamine Hagedorn (NPH) units. She denied using NSAIDs or herbal supplements. On presentation, her vital signs were a temperature of 36.4°C, heart rate of 86/minute, blood pressure of 138/79 mmHg, RR of 16/minute, and oxygen saturation of 96% on room air. Her abdomen was soft with mild epigastric tenderness and no signs of peritonitis. The remainder of the physical exam was unremarkable.

Complete blood count (CBC), renal function, and serum electrolytes were within normal range. Serum glucose was elevated. Serum AST and ALT were elevated to 3-4 times upper limit of normal while ALP was elevated to 2-3 times upper limit of normal and total bilirubin was normal consistent with mixed pattern of liver injury. However, R factor was 1.5, suggesting cholestatic liver injury. She had high CRP and gamma-glutamyl transpeptidase. Serum ACE, 25 hydroxy vitamin D, and 1.25 hydroxy vitamin D were normal. Serum antismooth muscle (Sm) antibody, antinuclear antibody (ANA), and antimitochondrial antibody (AMA) were negative.  Table 1 summarizes laboratory findings with abnormal values bolded. Urinalysis was unremarkable. Stool exam for ova and parasites, bacterial culture, and C diff. toxin PCR was negative. Schistosoma IgG antibody via enzyme immunoassay was negative. Viral hepatitis panel and HIV testing were negative. QuantiFERON TB Gold test was negative.

**Figure 1:**
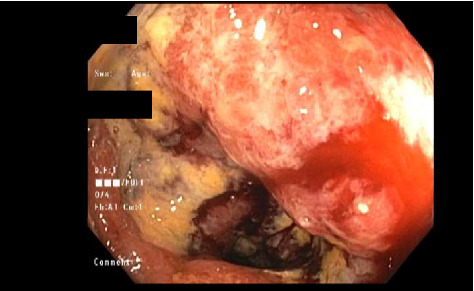
Colonoscopy. Colonoscopy showing severe diffuse erythema, hyperemia, and ulceration in ascending colon just proximal to hepatic flexure consistent with severe colitis.

**Figure 2:**
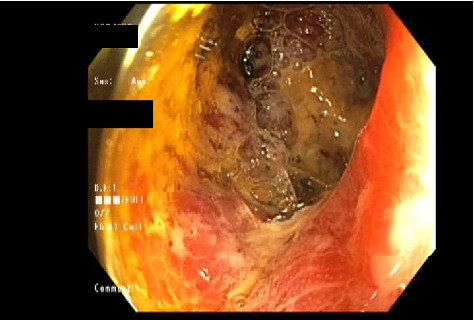
Colonoscopy. Colonoscopy showing severe diffuse erythema, hyperemia, and ulceration in ascending colon just proximal to hepatic flexure consistent with severe colitis.

**Figure 3:**
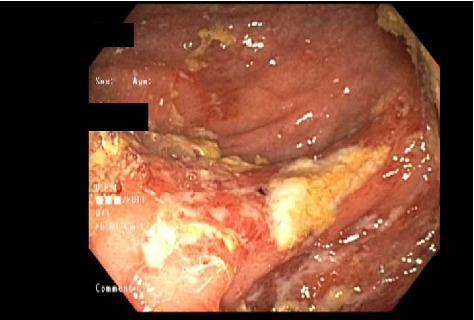
Colonoscopy. Colonoscopy showing severe diffuse erythema, hyperemia, and ulceration in ascending colon consistent with severe colitis.

**Figure 4:**
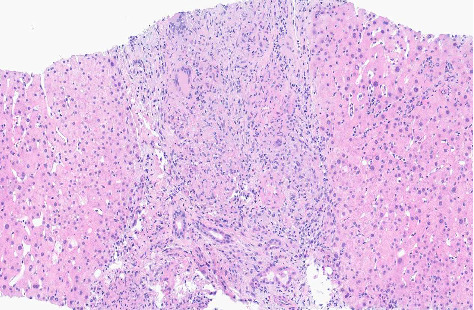
Hematoxylin and eosin stain liver biopsy (10× magnification). Liver biopsy shows portal tracts containing moderate mixed inflammatory cell infiltrates comprised predominantly lymphocytes, with few admixed eosinophils, neutrophils, and plasma cells. No lobular necro inflammatory activity is evident. Native bile ducts are intact with rare mild ductular reaction with intraepithelial neutrophils. There is mild macro vesicular steatosis in a centrilobular distribution. Ballooned hepatocytes are not identified. Additionally, portal tracts containing large poorly formed portal noncaseating granulomas with multinucleated giant cells are seen. Not shown in image but trichrome and reticulin stains revealed no fibrosis, and AFB and GMS stains reveal no mycobacteria or fungal organisms, respectively.

**Figure 5:**
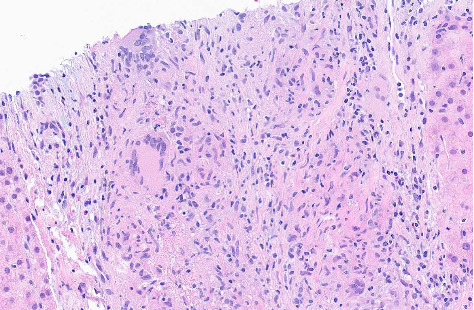
Hematoxylin and eosin stain liver biopsy (20× magnification). Liver biopsy shows portal tracts containing moderate mixed inflammatory cell infiltrates comprised predominantly lymphocytes, with few admixed eosinophils, neutrophils, and plasma cells. No lobular necro inflammatory activity is evident. Native bile ducts are intact with rare mild ductular reaction with intraepithelial neutrophils. There is mild macro vesicular steatosis in a centrilobular distribution. Ballooned hepatocytes are not identified. Additionally, portal tracts containing large poorly formed portal noncaseating granulomas with multinucleated giant cells are seen. Not shown in image but trichrome and reticulin stains revealed no fibrosis, and AFB and GMS stains reveal no mycobacteria or fungal organisms, respectively.

**Figure 6:**
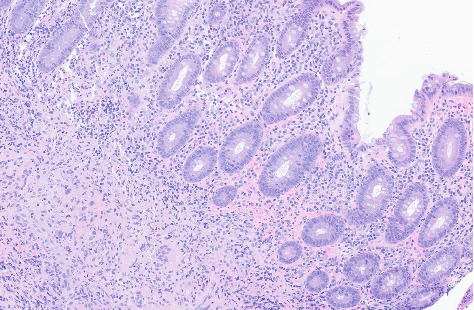
Hematoxylin and eosin stain ascending colon biopsy (10× magnification). Colon biopsy shows focal ischemic injury and active colitis. Multiple fragments containing epithelioid granulomas are seen in mucosa and submucosa. Not shown in image but GMS and AFB stains were negative for fungal elements and acid-fast bacilli, respectively.

**Figure 7:**
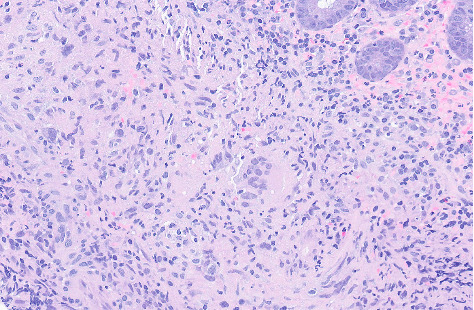
Hematoxylin and eosin stain ascending colon biopsy (20× magnification). Colon biopsy shows focal ischemic injury and active colitis. Multiple fragments containing epithelioid granulomas are seen in mucosa and submucosa. Not shown in image but GMS and AFB stains were negative for fungal elements and acid-fast bacilli, respectively.

CT scan of the chest was unremarkable. CT scan of the abdomen and pelvis revealed mild prominence of extrahepatic bile ducts and colonic wall thickening, affecting the cecum and ascending colon with adjacent slight fat stranding. Mesenteric Doppler showed normal flow and perfusion. MRCP showed nonspecific enlarged periportal lymph nodes, mild intrahepatic and common bile duct dilatation (CBD), and peribiliary enhancement concerning cholangitis. Esophagogastroduodenoscopy (EGD) with endoscopic ultrasound (EUS) showed a normal upper endoscopic exam and a dilated CBD to 9 mm with no stones, sludge, or strictures. Her CBD dilation was attributed to her previous cholecystectomy. Colonoscopy (Figures 1, 2, and 3) revealed a diffuse area of severely erythematous, hyperemic, and ulcerating mucosa in the ascending colon just proximal to the hepatic flexure at about 80 cm, the remainder of the colon appeared normal due to the risk of perforation scope was not advanced further to the ileocecal valve, cecum, and terminal ileum. Liver biopsy (Figures 4 and 5) showed large portal noncaseating granulomas, and the Gomori methenamine silver (GMS) and acid-fast bacilli (AFB) stains were negative. Random gastric and duodenal biopsy returned normal. Colon biopsy (Figures 6 and 7) from the inflamed segment showed active inflammation with focal areas of ischemia and ulceration; multiple fragments contained epithelioid granulomas in the submucosa and mucosa. Special stains such as GMS and AFB were negative for fungal elements and AFB.

To summarize, the case presented an elderly female with abdominal pain without diarrhea, elevated liver function tests (LFTs), and severe right-sided colitis. There was no evidence of fever, jaundice, or leukocytosis; blood cultures were negative, and EGD with EUS was normal, excluding biliary causes of elevated LFTs. A negative ANA, anti-Sm, and AMA excluded primary biliary cholangitis and autoimmune hepatitis. The finding of noncaseating granulomas on liver biopsy resembled hepatic sarcoidosis. Regarding colitis, lack of bloody diarrhea or hypotension, normal lactic acid, and mesenteric duplex vascular ultrasound excluded ischemic etiology. Normal stool culture, Schistosoma antibodies, negative AFB, and GMS stains helped exclude common infectious causes of granulomatous colitis. IBD, especially Crohn's disease (CD) and sarcoidosis, remained as a differential for colitis. The age of presentation was atypical for both diseases; she did have normal ACE levels, 1,25 hydroxy Vitamin D levels, and thoracic imaging. However, symptom profile and lack of chronic diarrhea, previous normal colonoscopy, lack of extraintestinal features of IBD, and presence of hepatic sarcoidosis pointed toward colonic sarcoidosis being the leading diagnosis. Moreover, abundant noncaseating granulomas are rarely seen on intestinal biopsies of adults with IBD.

Prior to most laboratory investigations, the patient was initially treated with empiric broad-spectrum antibiotics with no response. She was treated with oral prednisone 40 mg daily, subcutaneous methotrexate 12.5 mg weekly, and oral ursodeoxycholic acid (UDCA) 500 mg twice daily with plans to taper prednisone during outpatient follow-up. We saw the patient in clinics at 4 weeks and 3 months following discharge. Her abdominal pain and nausea improved dramatically, and oral intake returned to normal within 4 weeks. Her LFTs normalized in 3 months with AST of 29 (8–37 U/L), ALT of 29 (8–35 U/L), ALP of 68 (50–150 U/L), and total bilirubin of 0.3 (0.1–1.0 mg/dL). Serum CRP became normal at < 4 (< 5 mg/L) in 3 months. Fecal calprotectin returned normal at < 50 mcg/g, and CT enterography showed resolved right-sided colon thickening and colitis with, otherwise, unremarkable exam for small and large bowel. Her outpatient rheumatologist tapered oral prednisone to 20 mg daily, and she remains on weekly injectable methotrexate without any signs of intolerance or side effects.

## 3. Discussion

Sarcoidosis is a multisystemic disease of unknown etiology characterized by infiltration of various organs with noncaseating epithelioid granulomas [1]. Although it affects individuals of any ethnicity or age, African Americans, Scandinavians, and individuals in the age group of 30–50 years are at the highest risk [1]. The estimated prevalence ranges from 2.17 to 160 per 100,000 individuals [1]. Most patients are asymptomatic, although 30% may experience nonspecific symptoms such as fatigue, fever, or weight loss, and organ-specific symptoms can also occur [1]. While the lungs are commonly affected, other organs, including the skin, eyes, heart, kidneys, central nervous system, musculoskeletal system, and GI tract, can also be involved [2]. Diagnosis primarily involves a biopsy of lymph nodes or affected organs demonstrating noncaseating epithelioid granulomas. Elevated ACE levels and 1,25 Vitamin D with hypercalcemia can support the diagnosis but are unnecessary [2]. Most patients with hilar lymphadenopathy, asymptomatic lung involvement, and normal pulmonary function tests require only observation [3]. However, symptomatic pulmonary involvement, abnormal pulmonary function test, symptomatic liver involvement, or involvement of other extrapulmonary organs warrants treatment [3]. Glucocorticoids and various immunosuppressants are used for treatment initially based on the severity of the organ affected and individual risks [3].

Liver involvement occurs in approximately 80% of the cases [4]. Hepatic sarcoidosis can lead to elevated alkaline phosphatase, hepatomegaly, portal hypertension, and chronic cirrhosis [4]. Splenic involvement with splenomegaly can also occur [5]. However, GI tract involvement is sporadic, occurring in only 0.1%–1.6% of the cases [6]. A study involving over 300 patients with GI sarcoidosis revealed various manifestations, with esophageal involvement in 14% of the cases causing dysphagia, gastric involvement in 51% causing diffuse infiltration and epigastric pain, and small bowel involvement in 17% causing obstruction, inflammation, ulceration, and rarely protein-losing enteropathy [7]. Rare reports of appendicular sarcoidosis (3% of the cases) presenting as perforation or rectal sarcoidosis (5% of the cases) presenting as a mass have also been documented [7]. Colonic sarcoidosis occurred in 10% of the cases [7].

Colonic sarcoidosis presents with varying symptoms, including abdominal pain, hematochezia, iron deficiency anemia, constipation, and diarrhea [7]. Endoscopic features can be diverse, ranging from single/multiple ulcers, friable mucosa mimicking colitis, plaque-like lesions with segmental narrowing, stenotic obstructive lesions, and numerous submucosal lesions mimicking malignancy [7]. Most reported presentations were as colonic polyps or obstructing mass with abnormal FDG PET activity [8–11]. Some studies suggest sarcoid-like reactions occur in patients after treatment of colon cancer. However, the evidence that sarcoidosis increases the risk of colon cancer is inconclusive [12].

Sarcoidosis has granulomas formed by activated macrophages, epithelioid cells, and multinucleated giant cells lacking central necrosis or caseation. The inability of macrophages to eliminate triggering antigens leads to the persistence of granulomas. Diagnosis of sarcoidosis heavily relies on this histopathologic finding in the setting of clinical suspicion. However, granulomatous lesions of the colon can occur from diverse etiologies. Infections from bacteria such as *Mycobacterium tuberculosis*, Yersinia, Syphilis, Bartonella, Salmonella, Whipple and Lymphogranuloma venereum, fungal organisms such as Histoplasma, Cryptococcus, Coccidiomycosis, and Basidiobolomycosis along with parasites such as Leishmaniasis, Schistosomiasis, and Anisakiasis can cause granulomatous colitis [13]. Stool culture or DNA PCR, Ziehl–Neelsen, Periodic Acid Schiff, and GMS stains aid in excluding infectious etiologies. Vasculitis such as granulomatosis with polyangiitis and eosinophilic granulomatosis with polyangiitis, foreign body reaction, and ischemic injury are other important causes.

The two most common and often inseparable causes include CD and sarcoidosis. Differentiating both poses a significant challenge to clinicians. Both carry an immune-mediated pathogenesis with the activation of T cells and upregulation of various interleukins and TNF alpha. Moreover, environmental factors, mycobacterial infection, and genetic susceptibility play an essential role in the pathogenesis of both conditions. A genome-wide association study revealed a statistically significant common single-nucleotide polymorphism (SNP) rs1398024 on chromosome 10p12.2 in patients with CD and sarcoidosis, raising questions about potential coexistence and overlap in genetically susceptible individuals [14, 15]. GI symptoms are comparable in both conditions. Many extraintestinal manifestations, such as erythema nodosum, arthralgia, and uveitis, are common in both diseases. Subtle differences exist, such as CD rarely involves hilar lymph nodes and lungs; granulomas are more prominent in sarcoidosis than CD, whereas transmural inflammation and fistula formation are unique to CD. Antisaccharomyces cerevisiae antibodies are elevated in CD, whereas sarcoidosis has hypercalcemia and elevated 1,25 Vitamin D and ACE levels; these are unreliable for concrete differentiation as many cases of sarcoid have normal 1,25 Vitamin D and ACE levels. Currently, clinicians rely on the index of suspicion and the presence of sarcoid granulomas in organs that are atypical for CD. Treatment with steroids, hydroxychloroquine, immunosuppressants, and biologics has been studied well for pulmonary, joint, skin, cardiac, renal, and neurologic sarcoidosis. However, limited data exist for treating GI tract sarcoidosis. Although most cases of colonic sarcoidosis improved with steroids, methotrexate was needed to sustain improvement in some cases [16, 17].

In most cases documented in the literature, symptoms typically arise in middle-aged individuals. However, our patient exhibited symptoms at an unusually advanced age. Additionally, isolated severe right-sided colitis as a presentation of colonic sarcoidosis is rare. We excluded ischemic or infectious causes of granulomatous colitis after a thorough investigation. Although CD can manifest in elderly individuals, the milder severity of GI symptoms and the presence of hepatic noncaseating granulomas on biopsy lean toward a diagnosis of colonic sarcoidosis rather than CD. While there is some overlap in treating both conditions, overall medical management differs significantly. The first case of colonic sarcoidosis was reported in 1949 [18]. Yet, even after 75 years, distinguishing between CD and GI tract sarcoidosis remains challenging. Conversely, research in recent decades has advanced our understanding of sarcoidosis pathophysiology. With the emergence of whole genome and exome sequencing, genetic markers could play a crucial role in accurately distinguishing between the two conditions or even demonstrating their coexistence. Managing the latter scenario requires a multidisciplinary approach involving gastroenterologists and rheumatologists. The existing knowledge gap underscores the necessity for developing new guidelines to address accurate differential diagnosis between CD and sarcoidosis, determine the efficacy and choice of biologics for treating GI tract sarcoidosis, and manage patients in cases of coexistence.

## Figures and Tables

**Table 1:** Summary of laboratory findings.

Laboratory test name (serum)	Measured value	Reference range
White blood cell count	5 × 10^3^/μL	3.5–11 × 10^3^/μL
Hemoglobin	13.1 g/dL	11.5–15.5 g/dL
Hematocrit	42.1%	36%–47%
Platelet count	303 × 10^3^/μL	150–450 × 10^3^/μL
Glucose	**179 mg/dL**	**60–99 mg/dL**
Sodium	138 mEq/L	135–145 mEq/L
Potassium	3.7 mEq/L	3.5–5 mEq/L
Chloride	100 mEq/L	96–106 mEq/L
Bicarbonate	25 mEq/L	22–28 mEq/L
Blood urea nitrogen	14 mg/dL	7–20 mg/dL
Creatinine	0.8 mg/dL	0.5–1.4 mg/dL
Corrected calcium	9.8 mg/dL	8.4–10.2 mg/dL
Phosphorous	3 mg/dL	2.5–4.5 mg/dL
Magnesium	1.9 mg/dL	1.7–2.2 mg/dL
C-reactive protein	**89 mg/L**	**< 5 mg/L**
Lactic acid	0.7 mmol/L	0.5–2 mmol/L
Lipase	24 U/L	11–65 U/L
Aspartate aminotransferase	**120** **U/L**	**8–37** **U/L**
Alanine aminotransferase	**145** **U/L**	**8–35** **U/L**
Alkaline phosphatase	**409** **U/L**	**50–150** **U/L**
Total bilirubin	0.5 mg/dL	0.5–1 mg/dL
Total protein	6.4 mg/dL	6–8.3 mg/dL
Albumin	**3.2 mg/dL**	**3.5–5 mg/dL**
Gamma-glutamyl transpeptidase	**246** **U/L**	**11–63** **U/L**
Angiotensin converting enzyme	60 U/L	16–85 U/L
25 Hydroxy vitamin D	22 ng/mL	20–99 ng/mL
1, 25 Hydroxy vitamin D	44 pg/mL	18–78 pg/mL

*Note:* The bold values show elevated glucose consistent with hyperglycemia, elevated CRP consistent with inflammation, low albumin, which is also due to inflammation, and elevated AST, ALT, ALP, and GGT consistent with abnormal liver tests as described in detail in manuscript.

Abbreviations: dL = deciliter, g = gram, L = liter, mEq = milliequivalent, mg = milligram, mL = milliliter, mmol = millimole, ng = nanogram, pg = picogram, U = units.
